# Diagnosis and treatment complications of primary cardiac lymphoma in an immunocompetent 28-year old man: a case report

**DOI:** 10.1186/s12885-019-5405-y

**Published:** 2019-03-01

**Authors:** Maria Bonou, Chris J. Kapelios, Athanasios Marinakos, Stamatis Adamopoulos, Panagiotis Diamantopoulos, Periklis G. Foukas, Loukas Kaklamanis, Penelope Korkolopoulou, John Barbetseas, Nora-Athina Viniou

**Affiliations:** 1 Cardiology Department, Laiko General Hospital, 17 Agiou Thoma Street, 11 527 Athens, Greece; 2 Heart Failure and Transplant Unit, Onassis Cardiac Surgery Centre, Athens, Greece; 3 Hematology Unit, 1st Department of Internal Medicine, National and Kapodistrian University of Athens School of Medicine, Laiko General Hospital, Athens, Greece; 4 2nd Department of Pathology, National and Kapodistrian University of Athens School of Medicine, Attikon University Hospital, Athens, Greece; 5 Department of Pathology, Onassis Cardiac Surgery Centre, Athens, Greece; 6 1st Department of Pathology, National and Kapodistrian University of Athens School of Medicine, Athens, Greece

**Keywords:** DLBCL primary cardiac lymphoma, Imaging, Treatment complications

## Abstract

**Background:**

Primary cardiac lymphomas (PCL) represent extremely rare cardiac tumors which are accompanied by poor prognosis, unless they are timely diagnosed and treated.

**Case presentation:**

Herein we present a 28-year-old, immunocompetent man who presented to our hospital due to progressively worsening symptoms and signs of superior vena cava syndrome. Multi-modality imaging demonstrated a large intracardiac tumor, which was proven, by biopsy, to be a PCL. The patient received targeted chemotherapy which led to total remission of his disease, with no relapse over a 15-month follow-up period.

**Conclusions:**

Although PCLs are rare, they should always be kept in mind in the differential diagnosis of cardiac tumors. Timely diagnosis of PCLs and appropriate chemotherapy, alone or in combination with radiotherapy, seems to provide the best results.

## Background

Primary cardiac tumors are extremely rare, appearing in less than 0.1% of cases in a large series of 12,000 autopsies [1, 2]. Among these, primary cardiac lymphomas (PCL) also represent an extremely slight minority of approximately 1% [3]. PCLs are accompanied by a poor prognosis, unless they are treated in the early stages [4]. For this reason, timely diagnosis is imperative.

## Case presentation

Herein we present the case of a 28-year-old man, with a free medical history who presented to the allergology department of our hospital due to progressively worsening over the past 3 months facial oedema and erythema of the upper thorax markedly aggravated by bending forward.

**Fig. 1:**
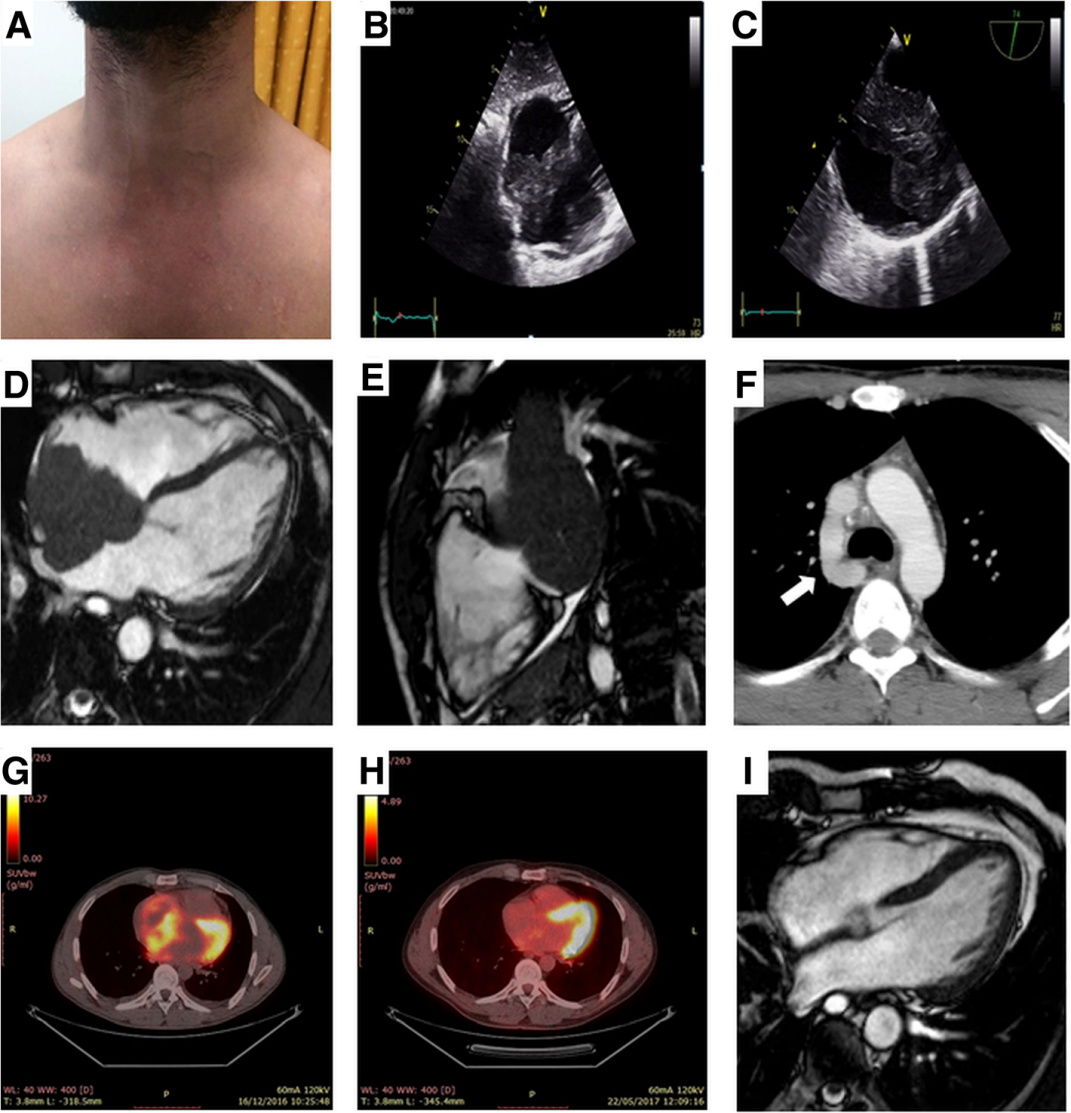
**a**. Dilated jugular veins and collaterals in the upper body. **b**. Transthoracic echocardiogram, subcostal view showing a large mass infiltrating the interatrial septum and extending mainly in the right atrium. **c**. Transesophageal echocardiogram, bicaval view showing a heterogeneous mass infiltrating the interatrial septum, filling almost three quarters of the right atrium, occluding the superior vena cava at its junction with the right atrium and extending into the left atrium. **d**. Cine MRI demonstrates a large infiltrating mass extending in both atria, occupying most of the right atrium, involving the surrounding pericardium leading to a mild pericardial effusion, and causing a grade of ostial stenosis of the lower right pulmonary vein. **e**. Cine MRI right ventricular long axis view with extension of neoplasm to the atriocaval junction and superior vena cava. **f**. Contrast-enhanced CT scan demonstrates a dilated azygos vein (arrow). **g**. 18-FDG PET/CT sagittal views showing increased 18-FDG-uptake within the tumor in the right atrium and **h**. complete tumor remission. **i**. Cine MRI four-chamber axial view showing no evidence of the tumor

At presentation, the patient demonstrated facial plethora with oedematous eyelids, dilated jugular veins and dilated chest wall collaterals (Fig. 1, panel a). The rest of his physical examination was unremarkable apart from bradycardia (50 beats per minute). From his laboratory findings at presentation marginally elevated c-reactive protein (CRP: 7.07 mg/l, normal values < 5), d-dimers (0.61 μg/ml), high-sensitivity troponin-T (18 pg/ml) and thyroid stimulating hormone (4.3 mU/l, normal values 0.17–4.05) were notable. The patient’s electrocardiogram revealed a coronary sinus rhythm, while the chest x-ray was unremarkable.

A transthoracic echocardiogram (TTE) depicted a large, non-mobile mass infiltrating the interatrial septum and extending to both atria, mainly to the right atrium (Fig. 1, panel b). The patient was admitted to the hospital and a transesophageal echocardiogram (TEE) demonstrated an heterogeneous mass infiltrating the interatrial septum, filling almost three quarters of the right atrium, which also occupied and occluding the superior vena cava at its junction with the right atrium (Fig. 1, panel c). On the following cine magnetic resonance imaging (MRI), with an improved visualization of the mass and its extension, the presence of the cardiac tumor was confirmed, also demonstrating infiltration of the surrounding pericardium, a mild pericardial effusion and obstruction of the superior vena cava by the tumor (Fig. 1, panel d). Mass extension was also noted in the left atrium causing some grade of ostial stenosis of the right pulmonary veins, while no lymph nodes were noticed (Fig. 1, panel e). The lesion was seen with heterogeneous high signal by T2-weighted imaging while a strong enhancement of the lesion was revealed during the late gadolinium phase.

**Fig. 2:**
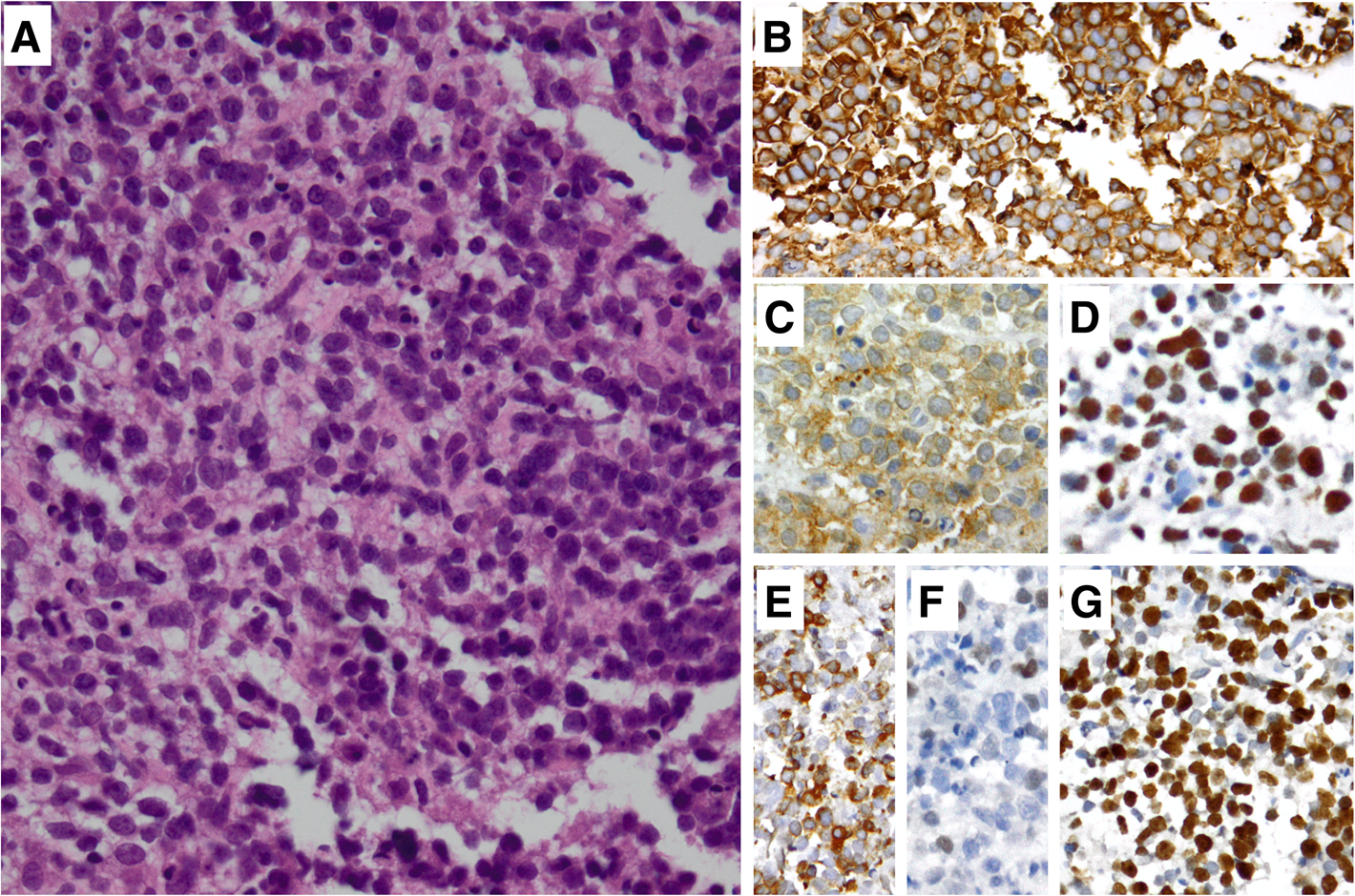
Microscopically (**a**, H&E stain), there was diffuse infiltration by medium-sized-to-large neoplastic lymphoid cells, that showed immunopositivity for CD20 (**b**), CD10 (**c**), Bcl-6 (**d**) and Bcl-2 (**e**), whereas only a small percentage was MUM1/IRF4+ (**f**). The Ki67 index was high (**g**) [magnification 40x for H&E (**a**) and for immunostains (**b**-**g**)]

A total body contrast-enhanced computed tomography (CT) scan demonstrated a dilated azygos vein (Fig. 1, panel f) with no extracardiac localizations of the disease. The occlusion of the superior vena cava was also confirmed by venography. A 18-fluorodeoxyglucose positron emission tomography/computed tomography (18-FDG PET/CT) revealed an abnormal hypermetabolic lesion which was confined to the heart, involving the right atrial cavity and the superior vena cava (Fig. 1, panel g). Further laboratory tests revealed serum levels of lactate dehydrogenase and β2-microglobulin within normal range. Bone marrow biopsy and immunophenotyping did not show abnormalities. The patient was subsequently submitted to an endomyocardial biopsy and the histopathological examination revealed diffuse large B-cell lymphoma (DLBCL, high grade Non-Hodgkin lymphoma, Fig. 2).

The patient was started chemotherapy with rituximab, cyclophosphamide, doxorubicin, vincristine and prednisolone (RCHOP). The first cycle drugs doses were divided in 2 with 15 days interval to minimize complications as arrhythmias and tissue rupture according to the literature [5]. Ten days after his first chemotherapy cycle the patient presented with severe rhythm disorders, pauses of up to 12 s, on the 24-h Holter monitoring accompanied by convulsions and a temporary pacemaker was implanted through the trans-femoral route. The rhythm disorders gradually disappeared as the chemotherapy treatment continued and the pacemaker was removed 10 days later. After four cycles of chemotherapy there was regression of the tumor on TTE, TEE and MRI (Fig. 1, panel i). However, the superior vena cava remained occluded, possibly due to fibrosis and thrombosis, and collateral vasculature was present. The treatment continued for another 4 cycles and at the end of chemotherapy, the patient underwent another 18-FDG PET/CT, which showed complete tumor remission (Fig. 1, panel h). Serial echocardiographic studies at 3, 6 and 12 and 15 months after completion of treatment confirmed the absence of relapse, as did a follow-up 18-FDG PET/CT at 15 months.

## Discussion and conclusions

Our patient represents one of the rare cases in which prompt diagnosis of PCL and initiation of appropriate treatment can lead to excellent clinical outcomes, despite the initial severe presentation.

PCLs are rare clinical entities which usually manifest after the fifth decade of life [6]. They are aggressive tumors, which are rapidly fatal, if left untreated [7]. For this reason, they must be timely differentiated from other, significantly more common cardiac tumors, such as myxomas and angiosarcomas. PCLs typically present in immunocompromised and HIV-positive patients, as well as in patients having undergone heart transplantation or receiving immunosuppressive medications [8]. However, approximately 25 cases of PCL in immunocompetent patients have also been described [9].

Macroscopically, PCL have been characterized as lymphomas only involving the heart and pericardium [8]. Histologically, most cases are DLBCL, although cases of anaplastic, plasmablastic and T-cell lymphomas have also been reported. Few data on cytogenetic alterations associated with PCL exist [8]; reports of bcl-2 expression, translocation (14;18) and an increased bcl-6 have been made [8].

PCLs typically present with dyspnea, arrhythmia or pericardial effusion [6]. The rarest presentation of all (approximately in 5%) is with symptoms and signs of superior vena cava syndrome [6], as in the case of our patient. Multi-modality imaging, including chest radiograph, echocardiography, CT, MRI and FDG PET/CT may be used for initial diagnosis and staging [10, 11]. However, limitations in imaging still exist [12], rendering endomyocardial biopsy a necessity for diagnosis.

Prognosis of patients with PCL is poor. Median survival after treatment is approximately 7 months [8]. Surgical resection of PCL is implemented only as bail-out strategy (and is associated with worse prognosis) or when less invasive procedures are insufficient to provide diagnosis [13, 14]. However, early surgery becomes first line treatment in cases when the tumor results in hemodynamical compromise, especially in tumors resistant to chemotherapy, as recently described [15]. In general, chemotherapy with CHOP (cyclophosphamide, doxorubicin, vincristine and prednisone) has been used in the majority of patients with reported PCL [6]. Patients are at risk of death early post chemotherapy due to massive pulmonary thromboembolism or tissue necrosis in cases with myocardial infiltration [8]. Decrease of the dose of cyclophosphamide and adriamycin in the initial course of chemotherapy may reduce the risk of death [8]. Substituting doxorubicin with etoposide has been accompanied with an increased risk of disease relapse, while the use of methotrexate, doxorubicin, cyclophosphamide, prednisone and bleomycin has been associated with a poor prognosis in T-cell PCL [8]. The addition of rituximab to CHOP has altered the natural history of DLBCL, as it has increased remissions and complete responses [4, 16]. The same applies for PCLs, which have shown significant improvement in prognosis following RCHOP introduction [8]. Radiotherapy use has also been reported in some patients, alone or adjuvant to chemotherapy [8].

Despite the rarity of PCLs, they should always be kept in mind in the differential diagnosis of cardiac tumors. Timely diagnosis of PCLs and chemotherapy with RCHOP, alone or in combination with radiotherapy, seems to provide the best results.

## Abbreviations

18-FDG PET/CT: 18-fluorodeoxyglucose positron emission tomography/computed tomography
CHOP: Cyclophosphamide, doxorubicin, vincristine and prednisone
CRP: C-reactive protein
CT: Computed tomography
DLBCL: Diffuse large B-cell lymphoma
MRI: Magnetic resonance imaging
PCL: Primary cardiac lymphoma
RCHOP: Rituximab, cyclophosphamide, doxorubicin, vincristine and prednisolone
TEE: Transesophageal echocardiogram
TTE: Transthoracic echocardiogram
